# NURSING HOME CLOSURES DURING THE COVID-19 PANDEMIC

**DOI:** 10.1093/geroni/igad104.1145

**Published:** 2023-12-21

**Authors:** Kelly Hughes, Micah Segelman, Kristie Porter, Miranda Diaz, Iara Oliveira

**Affiliations:** RTI International, Research Triangle Park, North Carolina, United States; RTI International, Washington, District of Columbia, United States; RTI International, Durham, North Carolina, United States; RTI International, Research Triangle Park, North Carolina, United States; Office of Behavioral Health, Disability, and Aging Policy (BHDAP), Washington, District of Columbia, United States

## Abstract

Nursing homes experienced unprecedented financial challenges during the COVID-19 pandemic, raising concerns about closures. This study identified closures from 2020-2021 using certification surveys and examined characteristics associated with closures using multivariate analysis. We interviewed nursing home industry experts and providers about pandemic-related challenges and policies affecting closure. Closures did not increase in 2020 and 2021 relative to 2011-2019. However, nursing homes with more staff shortages and COVID-19 infections were more likely to close. Additional characteristics associated with closure include smaller size, lower occupancy rate, and fewer Medicare residents. Interviews noted the following financial challenges: costly contract staffing, increased PPE and COVID-19 testing costs, reduced occupancy rates, and increased internal resources needed to implement new infection guidance. Interviews suggested that strategies to mitigate staffing shortages and federal and state monetary assistance may have prevented closures in 2020 and 2021, but continuing attention to closures is needed in 2022 and 2023 as funding ends.

